# Exploring the association between adherence to home-based exercise recommendations and recovery of nonspecific low back pain: a prospective cohort study

**DOI:** 10.1186/s12891-024-07705-6

**Published:** 2024-08-01

**Authors:** R. M. Arensman, M. F. Pisters, C. J.J. Kloek, T. Koppenaal, C. Veenhof, R. J.W.G. Ostelo

**Affiliations:** 1 Center for Physical Therapy Research and Innovation in Primary Care, Julius Health Care Centers, Utrecht, The Netherlands; 2 Physical Therapy Research, Department of Rehabilitation, Physiotherapy Science and Sport, Brain Center, University Medical Center Utrecht, Utrecht University, Utrecht, The Netherlands; 3 Research Group Empowering Healthy Behaviour, Department of Health Innovations and Technology, Fontys University of Applied Sciences, Eindhoven, The Netherlands; 4 Expertise Center Healthy Urban Living, Research Group Innovation of Human Movement Care, HU University of Applied Sciences, Utrecht, The Netherlands; 5 Department of Health Sciences, Faculty of Science, VU University Amsterdam, Amsterdam Movement Sciences Research Institute Amsterdam, Amsterdam, the Netherlands; 6 Department of Epidemiology and Data Science, Amsterdam University Medical Centre, Location Vrije Universiteit, Amsterdam, the Netherlands

**Keywords:** Exercise, Home-based exercise, Adherence, Low back pain, Disability

## Abstract

**Background:**

Adherence to home-based exercise (HBE) recommendations is critical in physiotherapy for patients with low back pain (LBP). However, limited research has explored its connection with clinical outcomes. This study examined how adherence to HBE relates to changes in physical function, pain intensity, and recovery from LBP in patients undergoing physiotherapy treatment.

**Methods:**

Data from a multicenter cluster randomized controlled trial in the Netherlands involving patients with LBP from 58 primary care physiotherapy practices were used. Adherence to HBE was assessed with the Exercise Adherence Scale (EXAS) at each treatment session. Previously identified adherence trajectories served as a longitudinal measure of adherence and included the classes “declining adherence” (12% of participants), “stable adherence” (45%), and “increasing adherence” (43%). The main outcomes included disability (Oswestry Disability Index), pain (Numeric Pain Rating Scale), and recovery (pain-free for > 4 weeks), which were measured at baseline and after three months. Linear and binomial logistic regression analyses adjusted for confounders were used to examine adherence–outcome relationships.

**Results:**

In the parent trial, 208 participants were included. EXAS scores were available for 173 participants, collected over a median of 4.0 treatment sessions (IQR 3.0 to 6.0). Forty-five (28.5%) patients considered themselves to have recovered after three months. The median changes in the Oswestry Disability Index and Numeric Pain Rating Scale were − 8 (IQR − 1 to -20) and − 2 (IQR − 0.5 to -4), respectively. The mean EXAS scores varied among patient classes: “declining adherence” (46.0, SD 19.4), “stable adherence” (81.0, SD 12.4), and “increasing adherence” (39.9, SD 25.3), with an overall mean of 59.2 (SD 25.3). No associations between adherence and changes in physical functioning or pain were found in the regression analyses.

**Conclusions:**

No association between adherence to HBE recommendations and changes in clinical outcomes in patients with LBP was found. These findings suggest that the relationship between adherence to HBE recommendations and treatment outcomes may be more complex than initially assumed. Further research using detailed longitudinal data combined with qualitative methods to investigate patient motivation and beliefs may lead to a deeper understanding of the relationship between adherence and clinical outcomes in patients with LBP.

## Introduction

Exercise therapy is often a primary choice physiotherapy treatment for patients with persistent nonspecific low back pain (LBP) [1]. It has also been shown to reduce pain and disability in patients with acute LBP [1]. Incorporating home-based exercise (HBE) into treatment plans can help alleviate the burden of LBP on the public health system. HBE, often recommended as a combination of strength and other exercises such as relaxation or postural exercises, has been shown to be effective in mitigating pain and disability in patients with LBP [2]. However, adherence to exercise recommendations is frequently low, with nonadherence rates reaching up to 70% in patients with LBP, which may substantially reduce the effectiveness of these interventions [3–5].

Studying patient adherence is a complex and challenging task because it is influenced by numerous external factors, such as financial constraints and healthcare accessibility, as well as patient-related factors, such as motivation and self-efficacy [6, 7]. Although external factors are beyond physiotherapist control, patient- and treatment-related factors can be effectively targeted through specific interventions [7]. Several factors have been linked to adherence, including physiotherapist guidance, the quantity of prescribed exercises, self-motivation, self-efficacy, past adherence behaviour, initial physical or aerobic activity levels, focus during exercise, increased pain during exercise, and significant levels of helplessness, depression, or anxiety [7, 8]. Additionally, adherence is not a static concept; it can vary over time. Distinct adherence trajectories have been observed in patients with LBP and osteoarthritis, indicating that adherence changes over time and that there are patient subgroups with similar patterns of adherence change [9, 10].

While there is evidence available identifying factors linked to adherence to HBE in patients with LBP [6, 11–13] and interventions aimed at enhancing adherence have been studied [14], the majority of adherence measurement tools either lack comprehensive psychometric testing or are too simplistic [15, 16]. Only in recent years have researchers developed and more rigorously tested novel measurement instruments, facilitating more detailed and long-term tracking of adherence to HBE recommendations in studies [17, 18].

In clinical practice, clinicians face the challenge of discerning whether to adjust their HBE recommendations due to ineffectiveness or whether they should provide additional support to their patients to enhance adherence when treatment effects fall short of expectations. Despite identifying different groups of patients with LBP and their distinct adherence trajectories over time as a potential solution, the fundamental assumption that adherence to HBE recommendations correlates with clinical outcomes remains insufficiently explored [10]. Consequently, the aim of this study was to explore the associations between adherence to HBE recommendations and changes in clinical outcomes in patients with LBP.

## Methods

### Study design

This study is a secondary cohort analysis using data from the e-Exercise LBP trial [19]. The e-Exercise LBP trial was a multicentre cluster randomized controlled trial investigating the effectiveness of a stratified blended physiotherapy intervention in patients with LBP [20]. Patients with LBP were recruited from 58 primary care physiotherapy practices in the Netherlands from January to June 2018. Patients received treatment from participating physiotherapists, and to avoid contamination between the intervention group and usual care group, physiotherapy practices were cluster-randomized to either the intervention group or usual care group. In the intervention group, physiotherapy consisted of face-to-face physiotherapy treatment combined with support from an eHealth application on their smartphone (e-Exercise LBP) [20, 21]. Patients in the usual care group received care based on the guidelines for LBP from The Royal Dutch Society for Physiotherapy [22]. The Medical Research Ethics Committee of the University Medical Center Utrecht, the Netherlands, approved the study (ISRCTN 94,074,203, registration date 20-07-2018).

### Participants

Patients were eligible for participation if they [1] requested physiotherapy treatment for LBP (pain in the lumbosacral region sometimes associated with radiating pain to the buttock or leg), [2, 22, 23] were aged 18 years or older, [3] had a smartphone or tablet with internet access, and [4] had B1-level proficiency in the Dutch language [24]. Patients were excluded if a specific cause of LBP (e.g. radiculopathy, ankylosing spondylitis, or skeletal metastases) was determined through medical imaging, if they were diagnosed by a medical doctor (including pelvic girdle pain caused by current pregnancy), or if they suffered from serious comorbidities. When inclusion for the trial ended, a total of 208 patients participated in the study.

### Outcomes

The outcomes measured were physical functioning, pain intensity, and recovery from LBP. The Oswestry Disability Index (ODI) version 2.1a [25, 26] was used to measure physical functioning. A higher ODI score (range 0-100) indicates increased physical disability. The ODI is included in the “Core Outcome Set” for research involving patients with nonspecific LBP [27]. The ODI change score was calculated by subtracting the ODI baseline score from the ODI score after three months. Pain intensity was measured using the Numeric Pain Rating Scale (NPRS) and was reported by the patient as an average score over the last seven days [26, 28]. If a patient experienced pain for fewer than seven days, the average pain intensity since the onset of pain was used instead. Pain scores on the NPRS range from 0 (no pain) to 10 (worst pain imaginable). The NPRS change score was calculated by subtracting the NPRS score at baseline from the NPRS score after three months. Recovery from LBP was determined based on patient self-reporting after three months. Recovery was defined as “being free from LBP for a minimum duration of four consecutive weeks.” Patients who met this criterion were classified as having recovered, while those who did not were classified as not having recovered.

### Exposures

The exposure of interest was adherence to HBE recommendations and was assessed by the physiotherapist using the Exercise Adherence Scale (EXAS) during every physiotherapy treatment session [18]. The EXAS measures adherence to frequency, intensity, and quality of performance recommendations. First, the physiotherapist instructed the patient in the performance of the exercises for at home and recorded the recommended frequency and intensity. At the start of the following treatment session, the patient reported adherence to the HBE recommendations, and the physiotherapist rated the quality of performance of the exercises by the patient on a 5-point Likert scale (poor, moderate, reasonable, good, excellent) [18]. For each exercise, adherence was then calculated by expressing patient-reported adherence as a percentage of physiotherapist recommendations for frequency and intensity, and the resulting percentage was modified by the quality of performance rating [18]. The mean score for all exercises was calculated and resulted in an EXAS score for every treatment session following the first session where exercises were recommended. The EXAS score ranges from 100 (perfect adherence to HBE recommendations) to 0 (no adherence to HBE recommendations). After the last treatment session, the therapist recorded the number of treatment sessions. To obtain the overall mean EXAS score, all EXAS scores for the individual treatment sessions were averaged.

Trajectory classes of adherence in the cohort of patients in this study were established in a prior study by utilizing EXAS scores from individual treatment sessions [10]. Three distinct adherence classes were identified: “declining adherence” (12% of participants), “stable adherence” (45% of participants), and “increasing adherence” (43% of participants). The trajectory classes served as a metric for changes in adherence over time.

### Potential confounders

Potential confounders were selected by searching the literature for factors known to be associated with adherence from cross-sectional studies [4, 7, 29–32] and the clinical expertise of the authors. Based on this, the following potential confounders were included in the initial analysis: age, sex, height, weight, BMI, education level, duration of LBP prior to the start of treatment, fear avoidance, pain catastrophizing, central sensitization, self-efficacy, self-management ability, and health-related quality of life.

Fear avoidance beliefs were measured using the Fear-Avoidance Beliefs Questionnaire (FABQ) [33]. The FABQ score ranges from 0 to 96, with a higher score indicating stronger fear and avoidance beliefs regarding the effects of physical activity on LBP.

For the measurement of pain catastrophizing, the Pain Catastrophizing Scale (PCS) was used [34]. The PCS score ranges from 0 to 52, with higher scores corresponding to higher levels of pain catastrophizing.

The Dutch Central Sensitization Inventory (CSI) was used to assess central sensitization [35]. The CSI score ranges from 0 to 100, with higher scores corresponding to higher levels of central sensitization.

Self-efficacy was assessed with the General Self-Efficacy Scale (GSE Scale) [36, 37]. The GSE scale score ranges from 10 to 40, with higher scores corresponding to greater self-efficacy.

Self-management ability was measured using the Dutch language version of the short-form Patient Activation Measure (PAM 13-Dutch) [38]. The PAM 13-Dutch score ranges from 0 to 100, and a higher score corresponds to higher levels of self-management.

Health-related quality of life was assessed with the EuroQol-5D-5 L [39]. A higher score (range 0–1) corresponds to higher health-related quality of life.

Treatment group allocation in the e-Exercise LBP parent trial [19] was the last potential confounder of interest.

### Data analysis

Data preparation was performed using SPSS 27 (IBM Corp. Released 2020. IBM SPSS Statistics for Windows, Version 27.0. Armonk, NY: IBM Corp.). Descriptive statistics were used to report patient characteristics, utilizing means and standard deviations (SDs) for normally distributed data and medians with interquartile ranges (IQRs) for data that were not normally distributed. Differences between adherence trajectory classes were assessed using ANOVA for normally distributed continuous outcomes and exposures, with the exception of the mean EXAS score. The EXAS score was excluded because the trajectory classes were derived from the EXAS, inherently maximizing differences between the classes [10]. For non-normally distributed data, the Kruskal-Wallis test was applied. Differences in the proportion of recovered patients between the classes were evaluated using the Chi-square test. Potential confounders were not assessed for differences between adherence trajectory classes, as a previous study had already evaluated this and found no significant differences between the classes [10]. Subsequent analyses were performed using R (R Foundation, Vienna, Austria) and an α of 0.05 was used for all significance tests. For an EXAS score, a minimum of two treatment sessions are required, which excluded patients with only one treatment session from the analyses. Multivariate imputation by chained equations was used to impute missing data in R using the ‘mice’ package, and an imputed dataset was generated for every percentage of cases with missing data [40, 41]. A case was labelled as “case with missing data” if a single data point or observation was missing for a participant, regardless of which data point or observation was missing. In total, 3.93% of all data points was missing, resulting in 52% cases with at least one missing data point. Consequently, 52 imputed datasets were created. The analyses and computations of the pooled results were performed on all imputed datasets using the ‘miceafter’ extension package for ‘mice’.

Linear regression and binomial logistic regression were used to test the relationship between adherence and the outcomes. The changes in the ODI and NPRS between baseline and after three months and recovery from LBP were used as outcomes. The mean EXAS score over all treatment sessions and the previously determined trajectory of adherence classes were used as determinants of adherence. Since the trajectory classes were determined using the EXAS scores of the same cohort of patients with LBP [10], only one of the adherence outcomes could be included in the regression models at a time. Therefore, all three outcomes were modelled using both determinants of adherence separately and adjusted for confounding factors, resulting in two models per outcome. To explore potential confounders, the association between the outcome and adherence was estimated with and without the potential confounders in the model. When the estimate of the association changed by more than 10%, the variable was added to the final model as a confounder. Furthermore, treatment group allocation from the parent trial was always included in the final model to control for the influence of the e-Exercise LBP intervention on adherence and outcomes [19]. For each regression model, the assumptions of linearity, homoscedasticity, independence and normality were checked and confirmed. Multicollinearity was assessed for the final models but was not found.

## Results

A total of 208 participants were included in the parent trial [19]. EXAS scores and trajectory of adherence class allocation were available for 173 participants who received two or more treatment sessions. The data were collected during a median of 4.0 treatment sessions [IQR 3.0, 6.0]. Missing data were caused by incomplete case reports forms or the absence of case reports forms from the participating physiotherapists, and loss to follow up (14 patients). Demographic characteristics of the included patients can be found in Table 1. After three months, forty-five (28.5%) patients considered themselves to have recovered from LBP. The median changes in the ODI and NPRS were − 8 [IQR − 20, -1] and − 2 [IQR − 4, -0.5], respectively. The mean EXAS score for all patients was 59.2 (SD 25.3), with 46.0 (SD 19.4) for the “declining adherence” class, 81.0 (SD 12.4) for the “stable adherence” class, and 39.9 for the “increasing adherence” class (SD 25.3). None of the outcomes or exposures assessed for differences in means, medians, or proportions between the three trajectory classes showed statistically significant differences.

**Table 1:** Demographic characteristics of the participating patients with low back pain. (*n* = 173)

Variable	Overall	“Declining adherence” class	“Stable adherence” class	“Increasing adherence” class
Number of patients	173	21	78	74
Age (years), median [IQR]	47.7 [35.2, 59.5]	45.0 [39.0, 56.8]	47.7 [35.9, 61.4]	49.1 [34.7, 56.0]
Sex (female), n (%)	85 (49.1)	11 (52.4)	35 (44.9)	39 (52.7)
Height (cm), mean (SD)	175.5 (9.7)	175.0 (7.3)	176.1 (9.7)	175.1 (10.3)
Weight (kg), median [IQR]	80.0 [70.0, 90.0]	80.0 [73.0, 85.0]	77.5 [67.2, 90.0]	79.0 [71.0, 93.0]
BMI, median [IQR]	25.5 [23.4, 28.1]	25.1 [23.4, 28.7]	24.9 [23.0, 27.7]	26.0 [23.7, 28.6]
**Education level, n (%)**				
low	30 (17.3)	5 (23.8)	15 (19.2)	10 (13.5)
middle	60 (34.7)	7 (33.3)	25 (32.1)	28 (37.8)
high	83 (48.0)	9 (42.9)	38 (48.7)	36 (48.6)
**Duration of LBP (weeks), n (%)**				
0–6 weeks	72 (41.6)	9 (42.9)	29 (37.2)	34 (45.9)
6–12 weeks	26 (15.0)	4 (19.0)	13 (16.7)	9 (12.2)
12 weeks-12 months	15 (8.7)	1 (4.8)	5 (6.4)	9 (12.2)
>12 months	60 (34.7)	7 (33.3)	31 (39.7)	22 (29.7)
ODI, median [IQR]	18.0 [8.0, 28.0]	22.0 [8.0, 34.0]	18.0 [10.0, 26.0]	18.0 [8.0, 28.0]
ODI change, median [IQR]	-8.0 [-20.0, -1.0]	-10.0 [-29.0, -1.0]	-8.0 [-16.0, -2.0]	-8.0 [-20.0, 0.0]
NPRS, median [IQR]	6.0 [4.0, 7.0]	6.0 [4.0, 7.0]	6.0 [4.0, 7.0]	6.0 [4.0, 7.0]
NPRS change, median [IQR]	-2.0 [-4.0, -0.5]	-2.0 [-4.0, 0.0]	-2.0 [-5.0, -0.8]	-3.0 [-4.2, -1.0]
FABQ, median [IQR]	23.5 [15.0, 33.0]	25.0 [18.0, 41.0]	21.0 [13.0, 31.0]	24.5 [17.0, 34.5]
PCS, median [IQR]	8.5 [4.0, 15.0]	12.0 [6.0, 15.0]	8.0 [4.0, 14.0]	9.5 [3.8, 17.0]
CSI, median [IQR]	27.0 [20.0, 38.0]	31.0 [20.0, 45.0]	26.0 [20.0, 36.0]	28.5 [20.0, 38.2]
GSE Scale, median [IQR]	33.0 [30.0, 35.8]	32.0 [30.0, 35.0]	34.0 [30.0, 36.0]	32.0 [30.0, 36.0]
PAM-13 Dutch, median [IQR]	63.1 [53.2, 72.5]	61.9 [52.7, 69.0]	63.1 [55.6, 72.5]	63.1 [53.2, 72.5]
EuroQol-5D-5 L, median [IQR]	0.9 [0.8, 1.0]	0.9 [0.8, 1.0]	0.9 [0.8, 1.0]	0.9 [0.8, 1.0]
Number of treatment sessions, median [IQR]	4.0 [3.0, 6.0]	6.0 [4.0, 7.0]	4.5 [3.0, 6.0]	4.0 [3.0, 7.0]
EXAS, mean (SD)	59.2 (25.3)	46.0 (19.4)	81.0 (12.4)	39.9 (25.3)
Recovered from LBP, n (%)	45 (28.5)	4 (21.1)	18 (25.4)	23 (33.8)
Treatment (intervention), n (%)	87 (50.3)	12 (57.1)	41 (52.6)	34 (45.9)

IQR Interquartile range, SD standard deviation, BMI Body Mass Index, LBP Low back pain, ODI Oswestry Disability Index, NPRS Numeric Pain Rating Scale, FABQ Fear-Avoidance Beliefs Questionnaire, PCS Pain Catastrophizing Scale, CSI Central Sensitization Inventory, GSE General Self-Efficacy, PAM-13 Dutch Patient Activation Measure, EXAS Exercise Adherence Rating Scale

The results from the linear regression analyses and the binomial logistic regression analyses can be found in Tables 2 and 3. The results from the analyses showed no statistically significant associations between determinants of adherence and changes in physical functioning or changes in pain when adjusted for confounders and controlling for the e-Exercise LBP intervention.

**Table 2:** Unadjusted models and adjusted models testing the relationship between adherence and changes in pain and disability. (*n* = 173)

Model	*n*	Unadjusted		Adjusted	
		Coefficient	95%-CI	Coefficient	95%-CI
*ODI change ~ EXAS*		0.07	-0.01–0.15	0.08	-0.00–0.17
*NPRS change ~ EXAS*		-0.00	-0.02–0.02	-0.00	-0.02–0.02
ODI change ~ “stable adherence”*	78	5.21	-1.50–11.92	4.58	-3.09–10.60
ODI change ~ “increasing adherence”*	74	4.38	-2.38–11.13	3.71	-2.20–11.36
NPRS change ~ “stable adherence”*	78	-0.38	-1.75–0.99	-0.39	-2.00–0.68
NPRS change ~ “increasing adherence”*	74	-0.49	-1.87–0.88	-0.48	-1.76–0.98

*95%-CI* 95% Confidence Interval, *ODI* Oswestry Disability Index after 3 months, *EXAS* Exercise Adherence Scale, *NPRS* Numeric Pain Rating Scale after 3 months. * “declining adherence” (*n* = 21) was used as the reference category. Models were adjusted for age, sex, body mass index, education level, Fear-Avoidance Beliefs Questionnaire score, Pain Catastrophizing Scale score, Central Sensitization Inventory score, General Self-Efficacy Scale score, Patient Activation Measure score, EuroQol-5D-5 L score, and treatment group. None of the models was statistically significant (*p* < 0.05)

**Table 3:** Unadjusted models and adjusted models testing the relationship between adherence and recovery. (*n* = 173)

Model	*n*	Unadjusted		Adjusted	
		OR	95%-CI	OR	95%-CI
*Recovery ~ EXAS*		1.01	1.00–1.02	1.02	1.00–1.03
Recovery ~ “stable adherence”*	78	0.78	0.23–2.68	0.82	0.23–3.00
Recovery ~ “increasing adherence”*	74	0.50	0.15–1.70	0.48	0.13–1.72

*OR* Odds Ratio, *95%-CI* 95% confidence interval, *Recovery* patient reported recovery from low back pain, *EXAS* Exercise Adherence Scale. * “declining adherence” (*n* = 21) was used as the reference category. Models were adjusted for age, sex, body mass index, education level, Fear-Avoidance Beliefs Questionnaire score, Pain Catastrophizing Scale score, Central Sensitization Inventory score, General Self-Efficacy Scale score, Patient Activation Measure score, EuroQol-5D-5 L score, and treatment group. None of the models was statistically significant (*p* < 0.05)

## Discussion

This study is among the first to explore the relationship between adherence to HBE recommendations and changes in clinical outcomes in patients with LBP. The results indicate that, both before and after adjusting for confounders, there are no significant associations between adherence to HBE recommendations and clinical outcome changes in LBP patients. Similarly, there is no evident association between LBP recovery and adherence. Although comparable literature focusing specifically on LBP patients is lacking, a similar study has examined the relationship between adherence to an HBE programme and clinical outcomes in patients with knee osteoarthritis [42]. A significant distinction from our study is the cross-sectional design used in the study. Nevertheless, the findings in patients with knee osteoarthritis are very similar to the findings of the current study. Although difficult to generalize, these findings suggest that there is no apparent association between adherence to exercise recommendations and changes in pain or disability or recovery from LBP.

Nevertheless, prior to confirming a lack of association between adherence and clinical outcomes, it is important to consider potential factors or underlying reasons that might account for these nonsignificant results. The first is that the construct of adherence to HBE recommendations is much more complex than previously thought. Existing research on predictors of adherence to HBE or other forms of exercise in patients with LBP reveals that patient factors, treatment-related factors, therapist factors, environmental factors, and social factors can influence adherence [3, 4, 6, 7, 11, 13, 30, 43–45]. Further complicating the construct of adherence is that the influence of these factors on adherence behaviour can differ significantly among patients. For example, reduced pain and disability from LBP as a result of HBE may encourage one patient to remain adherent, while another might discontinue exercising, believing it is unnecessary as their pain and limitations decrease. In contrast, increased pain may prompt one patient to cease exercising while stimulating another to exercise more. Unfortunately, because the outcomes were not measured during every physiotherapy session, this remains hypothetical. However, this could explain the large standard deviations of the mean EXAS scores for the different groups in the current study. Furthermore, although the regression models were adjusted for a number of factors (e.g., age, body mass index, education level, pain catastrophizing, self-efficacy, self-management), many factors could not be adjusted for.

A second explanation is that although adherence to frequency, intensity, and quality of performance recommendations are important indicators of adherence, the EXAS might not be optimal for their measurement. Despite improvements in existing measures of adherence, the accuracy of the EXAS score is limited by patient reporting bias (e.g., recall or patient honesty) and reporting errors by the physiotherapist. It appears that properly investigating the intricate connection between adherence to HBE recommendations and recovery from LBP calls for innovative research approaches. An initial step could involve gathering data on adherence and clinical outcomes during every treatment session and throughout the follow-up period, allowing comprehensive longitudinal analysis. Technological advances and innovations such as the TRAK© telerehabilitation tool [46] might lead to novel platforms to prescribe and support HBE and facilitate the measurement of adherence and clinical outcomes on a larger scale. By also integrating qualitative methods to explore patient motivations and beliefs, a more holistic understanding of adherence can be achieved. Emerging new insights might then contribute to the development of effective strategies for enhancing adherence in patients with LBP.

The third potential explanation is that despite the recommendation that exercise should be considered for routine use in the treatment of patients with persistent LBP, not all forms of exercise are effective [1, 47]. Furthermore, the treatment for this group of patients often requires additional treatment modalities such as cognitive behavioural therapy or interdisciplinary rehabilitation [1]. In the current sample, approximately 43% of patient experienced persistent LBP, which might have contributed to the lack of a statistically significant association between adherence to HBE recommendations and changes in clinical outcomes.

The last explanation is that the number of patients in some groups used in the regression models was relatively small, which reduces precision and might be the reason for the wide 95% confidence intervals (95% CIs). This is especially apparent for the models testing the relationship between adherence and recovery. Only 28.5% of participants considered themselves to have recovered from LBP after 3 months, which equates to 4 (21.1%) participants who recovered from the “declining adherence” trajectory class. The “increasing adherence” class had the highest percentage of recovered participants (33.8%) and had the largest effect (OR 0.48), with a wide 95% CI ranging from 0.13 to 1.72, indicating that a lack of precision might be the cause of the nonsignificant difference. This variability suggests an underlying trend that recovery is associated with the trajectory of the patient’s adherence class, although this finding was not statistically significant in this study. Therefore, while the current results do not establish a definitive statistical association, they do hint at a potential relationship that warrants further investigation in future studies with more participants to achieve narrower confidence intervals and more definitive conclusions.

This study has several important strengths. The data for this study were collected as part of a prospective, multicenter cluster randomized controlled trial, and the included patients reflected the characteristics of patients with LBP typically treated in primary care physiotherapy practices in the Netherlands [19, 48]. Therefore, the results from this study can be generalized to the population of patients with LBP in the Netherlands. Another strength is the use of multiple imputation to handle missing data. With 52% of the participants having at least one missing data point, performing complete case analyses would have severely limited the statistical power and reduced the robustness of the findings.

There are some limitations to the current study. First, although the EXAS provides data on adherence to HBE recommendations for every treatment session separately, the other outcomes in the study were assessed only at the start and after three months. This design limits the possibilities for repeated measures analysis, resulting in less precise regression models. However, measuring all outcomes at every treatment session leads to considerable additional administrative burden on patients, therapists, and researchers. Short and high-quality measurement instruments, such as those from the Patient-Reported Outcomes Measurement Information System (PROMIS^®^), might help mitigate those downsides [49].

A second limitation is that data on patient adherence were collected by physiotherapists. Although all participating physiotherapists were trained for data collection, adherence data from 21 patients were lost and could not be used in the analyses. However, given the current results, it is unlikely that without lost data, the analyses would have produced different results.

Ultimately, while increasing adherence may seem to be an easy solution to improve treatment effects, the results from the current study, along with the complexity of the construct of adherence and its measurement, suggest a more intricate relationship that warrants further investigation.

## Conclusions

This study explored the association between adherence to HBE recommendations and changes in clinical outcomes for patients with LBP. Contrary to expectations, no association was found between adherence measures and changes in clinical outcomes. These findings suggest that the relationship between adherence to HBE recommendations and treatment outcomes may be more complex than initially assumed. Further research using detailed longitudinal data combined with qualitative methods to investigate patient motivation and beliefs may lead to a deeper understanding of the relationship between adherence and clinical outcomes in patients with LBP.

## Data Availability

The datasets used and/or analysed during the current study are available from the corresponding author upon reasonable request.

## Abbreviations

LBP: Low Back Pain
HBE: Home-based Exercise
ODI: Oswestry Disability Index
NPRS: Numeric Pain Rating Scale
EXAS: Exercise Adherence Scale
FABQ: Fear-Avoidance Beliefs Questionnaire
PCS: Pain Catastrophizing Scale
CSI: Central Sensitization Inventory
GSE Scale: General Self-Efficacy Scale
PAM: Patient Activation Measure
IQR: Interquartile range
SD: Standard Deviation
95%-CI: 95% confidence interval
